# Soft Tissue Sarcoma of the Hand or Foot: Conservative Surgery and Radiotherapy

**DOI:** 10.1080/13577149977820

**Published:** 1999-03

**Authors:** Rema Jyothirmayi, Yoga Sittampalam, Clive Harmer

**Affiliations:** 1 Sarcoma Unit The Royal Marsden Hospital Fulham Road London SW3 6jj UK; 2 Department of Statistics The Royal Marsden Hospital Fulham Road London SW3 6jj UK; 3 Department of Radiotherapy The Royal Marsden Hospital Downs Road Sutton Surrey SM2 5PT UK

## Abstract

*Purpose.*  Conservative treatment in the form of limited surgery and post-operative radiotherapy is controversial in hand and foot sarcomas, both due to poor radiation tolerance of the palm and sole, and due to technical difficulties in achieving adequate margins.This paper describes the local control and survival of 41 patients with soft tissue sarcoma of the hand or foot treated with conservative surgery and radiotherapy. The acute and late toxicity of megavoltage radiotherapy to the hand and foot are described. The technical issues and details of treatment delivery are discussed. The factors influencing local control after radiotherapy are analysed.

*Subjects .*  Eighteen patients had sarcomas of the hand and 23 of the foot. All patients received post-operative radiotherapy, the majority receiving a dose of 60 Gy in 2-Gy daily fractions using a two-phase treatment.

*Results .*  The acute and late toxicity of treatment were within acceptable limits. The actuarial 5-year overall survival of the whole patient group was 67.6% and the local relapse-free survival was 44%.The local control was similar in tumours of hand and foot, and in patients treated at first presentation or relapse.

*Discussion.*  Post-operative radiotherapy to the hand or foot appears to be a well tolerated treatment resulting in long-term local control in a significant proportion of patients. The increased frequency of recurrence within the high-dose volume suggests the need for the use of higher total doses of radiotherapy.


*Sarcoma* (1999) 3, 17–24

ORIGINAL ARTICLE

### Soft tissue sarcoma of the hand or foot: conservative surgery and radiotherapy

REMA JYOTHIRMAYI,[1] YOGA SITTAMPALAM[2] & CLIVE HARMER[1]

[1]*Sarcoma Unit and* [2]*Department of Statistics, The Royal Marsden Hospital, Fulham Road, London SW3 6JJ, UK*

**Abstract**

*Purpose.* Conservative treatment in the form of limited surgery and post-operative radiotherapy is controversial in hand and foot sarcomas, both due to poor radiation tolerance of the palm and sole, and due to technical difficulties in achieving adequate margins. This paper describes the local control and survival of 41 patients with soft tissue sarcoma of the hand or foot treated with conservative surgery and radiotherapy. The acute and late toxicity of megavoltage radiotherapy to the hand and foot are described. The technical issues and details of treatment delivery are discussed. The factors influencing local control after radiotherapy are analysed.

*Subjects.* Eighteen patients had sarcomas of the hand and 23 of the foot. All patients received post-operative radiotherapy, the majority receiving a dose of 60 Gy in 2-Gy daily fractions using a two-phase treatment.

*Results.* The acute and late toxicity of treatment were within acceptable limits. The actuarial 5-year overall survival of the whole patient group was 67.6% and the local relapse-free survival was 44%. The local control was similar in tumours of hand and foot, and in patients treated at first presentation or relapse.

*Discussion.* Post-operative radiotherapy to the hand or foot appears to be a well tolerated treatment resulting in long-term local control in a significant proportion of patients. The increased frequency of recurrence within the high-dose volume suggests the need for the use of higher total doses of radiotherapy.

**Keywords**: *sarcoma, radiotherapy, hand, foot, conservative surgery.*

### Introduction

Conservative surgery combined with radiotherapy is the recommended treatment for soft tissue sarcoma of the extremity.[1–3] However, this approach is controversial in sarcoma of the hand or foot, due to difficulty in achieving wide excision margins and poor radiation tolerance of normal tissues. Resection alone results in a high local recurrence rate of 40–50%; thus amputation is usually recommended as the treatment of choice.[4–6] However, several recent reviews suggest that combined modality treatment with limited surgery plus radiotherapy can result in good local control with preservation of function.[7–13] Due to the rarity of soft tissue sarcoma in the distal extremities, most reviews include only a small number of patients, treated with a variety of radiation techniques. In addition most series include sarcomas of the wrist and ankle. The additional problem with the hand or foot is the low radiation tolerance of the skin of the palm and sole compared to the rest of the limb, and the limitation of salvage surgery for recurrence.

In this paper we retrospectively review 41 patients with soft tissue sarcoma arising from hands or feet treated with conservative surgery and post-operative radiotherapy. The acute and late toxicity of megavoltage radiotherapy, local control and functional outcome, as well as feasibility of salvage therapy, are analysed.

### Subjects and methods

### Patient characteristics

Between 1981 and 1997, 41 patients with soft tissue sarcoma arising from the hand or foot received radiotherapy at the Royal Marsden Hospital. All patients had non-metastatic disease and treatment was intended to be curative. The mean age was 39 years (range 13–76 years). Twenty-one patients were male and 20 female. Eighteen had tumours of the hand and 23 of the foot. Thirty-one patients were irradiated following surgery at initial presentation and 10 after surgery for local recurrence. Full history and clinical examination were performed. The surgical notes and pathology reports were reviewed. Investigations included chest X-ray and tomographic scans of the

Correspondence to: Rema Jyothirmayi, Department of Radiotherapy, The Royal Marsden Hospital, Downs Road, Sutton, Surrey SM2 5PT, UK. Tel: 0181 642 6011; Fax: 0181 643 5468.

1357-714X/99/010017-08 $9.00 © 1999 Taylor & Francis Ltd chest to exclude metastases. The histological diagnosis of sarcoma was confirmed in all patients. The patient and tumour characteristics are summarised in Table 1.

*Treatment details*

All patients underwent initial surgery, ranging from biopsy to wide local excision, depending on the location and size of tumour. Four patients underwent biopsy only. Fourteen underwent intracapsular excision (i.e. excision with macroscopic residual tumour), 12 marginal excision (i.e. excision of gross tumour with pseudo capsule) and 11 wide excision (excision of tumour with a margin of normal tissue, including 3 requiring ray amputation of a digit). Six patients underwent skin grafting as part of the initial surgical procedure. The patients who underwent suboptimal surgery were operated on in other hospitals including centres abroad, and were referred post-operatively for radiotherapy. The margins of excision were assessed from the histopathology report. Nineteen patients had clear excision margins and 3 had close margins. The excision margins were microscopically positive in 13 patients and 6 had gross residual tumour prior to radiotherapy.

All received post-operative radiotherapy. Twenty-six received conventional dose fractionation, ranging from 50 Gy in 25 fractions over 5 weeks to 60 Gy in 30 fractions over 6 weeks. Six patients received hyperfractionated radiotherapy to a dose of 72 Gy in 60 fractions of 1.2 Gy over 6 weeks, treating twice daily. Nine patients treated in the earlier part of the study were hypofractionated to a dose of 39.6 Gy in six fractions each of 6.6 Gy, given once a week.

The technique of radiotherapy was determined by the site and extent of tumour. Twenty-six patients were treated using a large phase I volume to a dose of 50 Gy in 25 fractions in the conventional group or 60 Gy in 50 fractions in the hyperfractionated group. This was followed by a smaller phase II volume adding 10 Gy in five fractions or 12 Gy in 10 fractions respectively during the sixth week. Fifteen patients received a single phase treatment throughout. All patients were treated with the limb immobilised in a cast (Figs 1 and 2).

Treatment portals were selected according to pre-operative computerised tomography (CT) scans and surgical notes. Irradiation of the entire circumference of the limb was avoided, with care being taken to spare a corridor of skin and subcutaneous tissue. The wrist and ankle joints were excluded from the high dose volume whenever possible. In patients with involvement of the foot, the sole was spared whenever possible. A direct electron beam of energy ranging from 5 to 10 MeV was used in 7 patients; photon beams of 5–6 MeV or Cobalt-60 were used in the remaining 34. Photon therapy techniques included parallel opposed or oblique wedged beams. The surgical scar required bolus to build up the skin dose in only 3 patients.

The overall treatment time ranged from 28 to 55 days (median 42 days). Two patients required treatment interruption due to an acute skin reaction. Six patients received chemotherapy as part of their initial therapy. The 3 patients with Ewing's sarcoma and three with rhabdomyo-sarcoma received regimes containing vincristine, actinomycin D, adriamycin and ifosfamide along with local radiotherapy. One patient with angiosarcoma had been treated with pre-radiotherapy intra-arterial adriamycin.

**Table 1**. *Patient characteristics*

| Factor | Number of patients | Percentage |
|---|---|---|
| Age at diagnosis (years) | | |
| 12–30 | 11 | 26.8 |
| >30–50 | 20 | 48.8 |
| >50 | 10 | 24.4 |
| Status at presentation | | |
| Primary | 31 | 75.6 |
| Recurrent | 10 | 24.4 |
| Gender | | |
| Male | 21 | 51.2 |
| Female | 20 | 48.8 |
| Site | | |
| Hand: Dorsum | 4 | 9.8 |
| Palm | 4 | 9.8 |
| Fingers | 10 | 24.4 |
| Foot: Dorsum | 12 | 29.3 |
| Sole | 7 | 17.1 |
| Medial aspect | 3 | 7.3 |
| Toes | 1 | 2.4 |
| Histology | | |
| Synovial sarcoma | 12 | 29.3 |
| Clear cell sarcoma | 7 | 17.1 |
| Epitheloid sarcoma | 6 | 14.6 |
| Rhabdomyosarcoma | 3 | 7.3 |
| Liposarcoma | 2 | 4.9 |
| Angiosarcoma | 2 | 4.9 |
| Others | 9 | 21.9 |

*Follow up evaluation*

Patients were followed up every 2 months for the first year and at longer intervals thereafter. Clinical examination and chest X-ray were performed at each visit. Further investigations were performed only when clinically indicated.

*Functional status and treatment toxicity*

The pre-radiotherapy functional status was retrospectively graded as normal, mild dysfunction or severe dysfunction (Table 2). Acute and late morbidity of radiotherapy were scored using the RTOG criteria. Limb oedema was scored using the NCIC late limb oedema scale. Function was also assessed at the time of last follow-up using the functional scales.

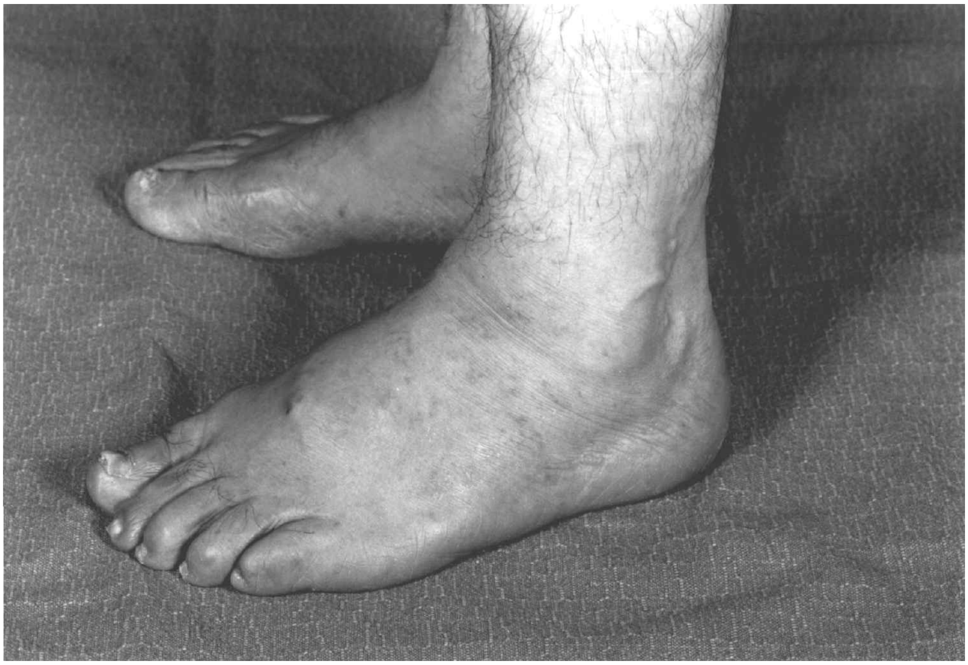

**Fig. 1.** *A 65-year-old man with a myxoid liposarcoma who presented with a large mass on the dorsum of the foot for which amputation was recommended. Tumour arose on the plantar aspect and was eroding two metatarsals.*

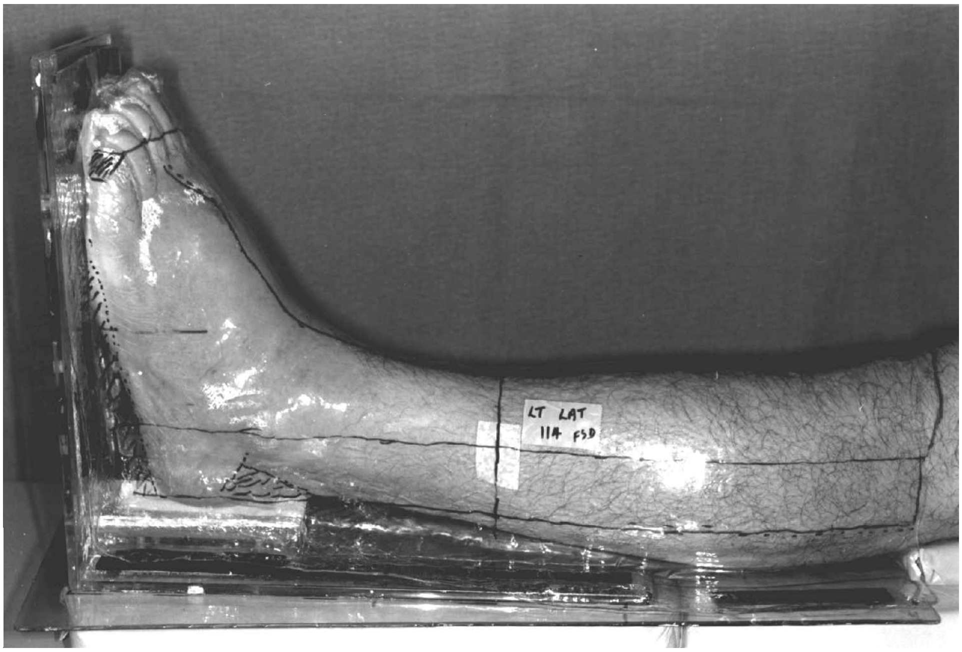

**Fig. 2.** *Left lateral field in perspex immobilisation cast of patient illustrated in Fig. 1. encompassing almost entire foot and flexor and extensor muscles up to the patella. This was opposed with a medial field to deliver a dose of 50 Gy to the 100% isodose in 25 fractions over 5 weeks, to resolve inhomogeneity. There is sparing of the terminal phalanges, sole of foot, Achilles tendon and a posterior corridor of normal tissue. A similar field arrangement was used for phase 2, but with fields confined to the foot.*

### *Statistical methods*

Survival analysis was performed using the Kaplan–Meier method and log-rank test. Analysis was done separately for overall survival and local recurrence. Survival was calculated from the date of surgery in all patients.

### Results

The median follow up was 65 months (range 3.5–205 months). Twenty-one patients had a completely normal pre-radiotherapy functional status; 13 had mild dysfunction and 7 severe dysfunction. All patients completed the planned course of radiotherapy, although 2 required treatment interruption due to acute toxicity. Thirty-nine patients had acute skin morbidity following radiotherapy with 14 patients having grade 1 reactions, 20 grade 2, and 5 grade 3 reactions. Two of the 6 patients with skin grafts developed ulceration of the graft which was managed conservatively, and healed without further surgical intervention.

**Table 2.** *Functional scale*

| | |
|---|---|
| Normal | Completely normal function and able to carry out all routine activities. No oedema or pain. |
| Mild dysfunction | Mild oedema of a limb, or some decrease in range of movement of a joint, or pain relieved by non-narcotic analgesic. |
| Severe dysfunction | Persistent moderate or severe oedema, or severe fibrosis, limitation of joint function, or pain requiring narcotic analgesic. |

Late morbidity, local control and survival were assessed in 34 patients treated prior to 1995, who had follow-up in excess of 30 months. The late toxicity of treatment is summarised in Table 3. No patient had persistent pain requiring analgesia.

Of these 34 patients, 14 (41%) developed local recurrence, of whom 9 had local recurrence alone and 5 had distant metastases in addition. Seven patients recurred within the phase II (radical dose) volume of whom 6 had either gross or microscopic disease. Five patients recurred in the phase I volume, and 2 patients had a marginal recurrence. Salvage treatment for local recurrence comprised wide re-excision in 8, ray amputation of digits in 3 and below knee amputation in 2, and 1 patient with inoperable recurrence underwent palliative re-irradiation. Four of 8 patients developed further local recurrence after wide re-excision. Of these, 3 had a further re-excision but 1 required a below elbow amputation. Four patients developed regional lymph node metastases of whom 2 had local recurrence in addition. Twelve patients developed distant metastases, of whom 6 had distant metastases alone, 5 distant and local relapse, and 1 distant and nodal relapse.

The median time to local recurrence was 19 months (range 4.5–85 months) and to distant metastases was 18 months (range 5–84 months). The pattern of failure in various potential prognostic groups is summarised in Table 4. High grade tumours had higher rates of both local and nodal failure (42 and 13% respectively). The margins of surgical excision did not appear to influence local control or metastatic rate, although these were assessed retrospectively from the pathological report in most cases. Histology of the primary tumour did not influence local control overall, although the subset of patients with Ewing's sarcoma or rhabdomyosarcoma had low rates of local recurrence (1/6 patients). Relapse rates were higher for patients treated for recurrence as compared to treatment at initial presentation.

**Table 3.** *Late radiation morbidity (RTOG criteria and NCIC late limb oedema scale)*

| Morbidity | | Number of patients | Percentage |
|---|---|---|---|
| Skin: | Grade I | 12 | 35.3 |
| | Grade II | 8 | 23.5 |
| | Grade II | 2 | 5.9 |
| Subcutaneous: | Grade I | 11 | 32.4 |
| | Grade II | 2 | 5.9 |
| Joints: | Grade I | 4 | 11.8 |
| | Grade II | 5 | 14.7 |
| Oedema: | Grade I | 9 | 26.5 |
| | Grade II | 1 | 2.9 |
| Bone: | Grade I | 3 | 8.8 |

Kaplan–Meier estimates showed an actuarial 5-year overall survival of 67.6% for the whole group (Fig. 3). Patients with hand sarcomas had overall survival of 73.3% compared to 63.6% for foot sarcomas (95% confidence interval, CI, 0.4–4.8) (Fig. 4).

Twenty-eight patients were free of disease at the time of last follow-up. Six patients were alive with disease (4 with distant metastases, 1 local and 1 nodal disease). Seven patients died of distant metastases. No patient died of uncontrolled local tumour.

The post-radiotherapy functional status was assessed at the time of last follow-up. No assessment could be made in the 2 patients who underwent extensive surgery for local recurrence. Of the remainder, 15 had normal function, 14 mild dysfunction and 3 had major dysfunction. The late cosmetic and functional outcome is shown in Figs 5 and 6.

### Discussion

Although large series have reported the results of conservative surgery and radiotherapy for extremity sarcomas, patients with involvement of the hand or foot are few in number.[1,14] A high incidence of late morbidity and functional deficits, often necessitating amputation, has been reported following irradiation.[15,16] In view of the low radiation tolerance of the skin of the palm and sole, radical resection, (often amputation), has been recommended as the primary treatment.[4,17] However, limb conservation therapy has gradually gained acceptance in this patient group. An improvement in radiation techniques and recognition of the importance of excluding the entire joint from the high-dose volume, plus sparing of a corridor of normal tissues, have resulted in reduced late morbidity from radiotherapy. The routine use of CT and magnetic resonance imaging (MRI) scans to define tumour volume more accurately and improved immobilisation techniques have resulted in a reduced high-dose planning target volume and field sizes in many patients. The use of three-dimensional planning and conformal radiotherapy may aid in further reduction of acute and late normal tissue damage.[18] There are a few reports of good local control with preservation of limb function using the combined modality approach which are summarised in Table 5.

**Table 4.** *Patterns of failure*

| Factor | Total number of patients | Number of patients with (%) Local failure | Nodal failure | Distant failure |
|---|---|---|---|---|
| Site of tumour | | | | |
| Hand | 12 | 5 (41.6) | 3 (25.0) | 2 (16.6) |
| Foot | 22 | 9 (41.0) | 1 (4.5) | 10 (45.5) |
| Age (years) | | | | |
| <45 | 17 | 9 (53.0) | 2 (10.5) | 7 (36.0) |
| >45 | 17 | 5 (29.4) | 2 (13.3) | 5 (33.3) |
| Gender | | | | |
| Male | 19 | 7 (36.8) | 2 (10.5) | 5 (26.3) |
| Female | 15 | 7 (46.6) | 2 (13.3) | 7 (46.6) |
| Histology | | | | |
| Synovial sarcoma | 11 | 7 (63.6) | 0 (0) | 5 (45.5) |
| Clear cell | 4 | 1 (25.0) | 3 (75.0) | 0 (0) |
| Epitheloid | 4 | 1 (25.0) | 0 (0) | 1 (25.0) |
| Tumour status | | | | |
| Primary | 26 | 10 (38.4) | 2 (7.7) | 10 (38.4) |
| Recurrent | 8 | 4 (50.0) | 2 (25.0) | 2 (25.0) |
| Grade | | | | |
| Low | 3 | 1 (33.3) | 0 (0) | 1 (33.3) |
| High | 31 | 13 (41.9) | 4 (12.9) | 11 (35.4) |
| Margins of excision | | | | |
| Negative | 16 | 6 (37.5) | 4 (25.0) | 5 (31.3) |
| Close | 3 | 2 (66.7) | 0 (0) | 3 (100) |
| Microscopic positive | 10 | 4 (40.0) | 0 (0) | 2 (20.0) |
| Gross tumour | 5 | 2 (40.0) | 0 (0) | 2 (40.0) |
| Radiotherapy | | | | |
| Conventional | 20 | 8 (40.0) | 4 (20.0) | 6 (30.0) |
| Hyperfractionated | 5 | 2 (40.0) | 0 (0) | 4 (80.0) |
| Hypofractionated | 9 | 4 (44.0) | 0 (0) | 2 (22.2) |

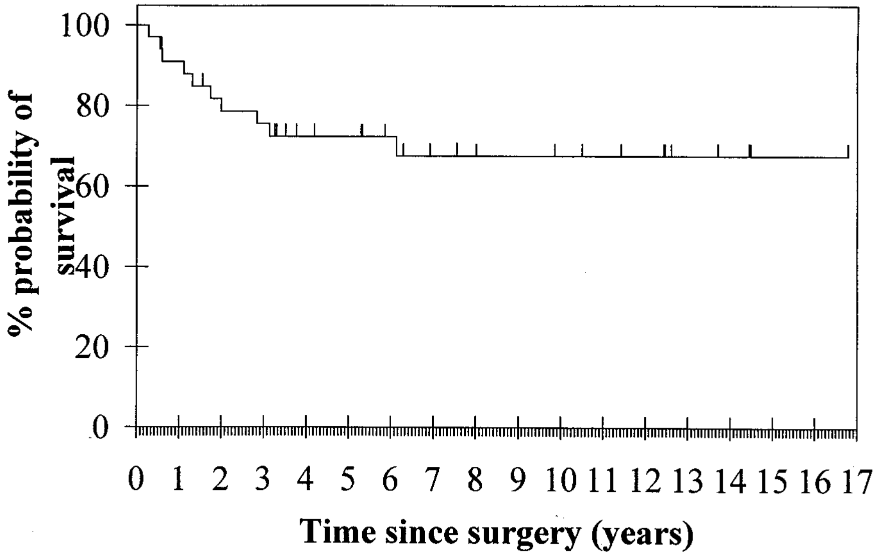

**Fig. 3.** *Graph showing 5-year overall survival of patients with hand and foot sarcomas.*

The usual recommended tumour dose for post-operative radiotherapy of soft tissue sarcoma is 60 Gy.[7] Twice daily fractionation has been proposed to improve local control by permitting dose escalation.[19] In our series, 6 patients received hyperfractionated radiotherapy to a total dose of 72 Gy over a period of 6 weeks. Acute toxicity was well tolerated although there was no apparent improvement in local control or survival.

Most studies demonstrate a higher rate of local failure in patients with positive surgical margins.[20,21] However, wide resection margins are difficult to achieve in sarcoma of the hand or foot due to anatomical constraints. The situation is analogous to

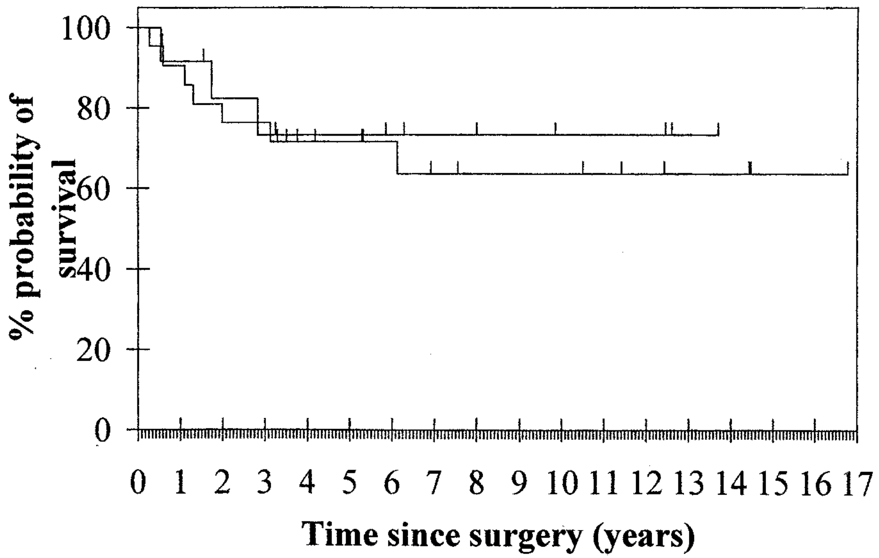

**Fig. 4.** *Five-year overall survival in patients with sarcomas of the hands in comparison with that of patients with sarcomas of the feet.*

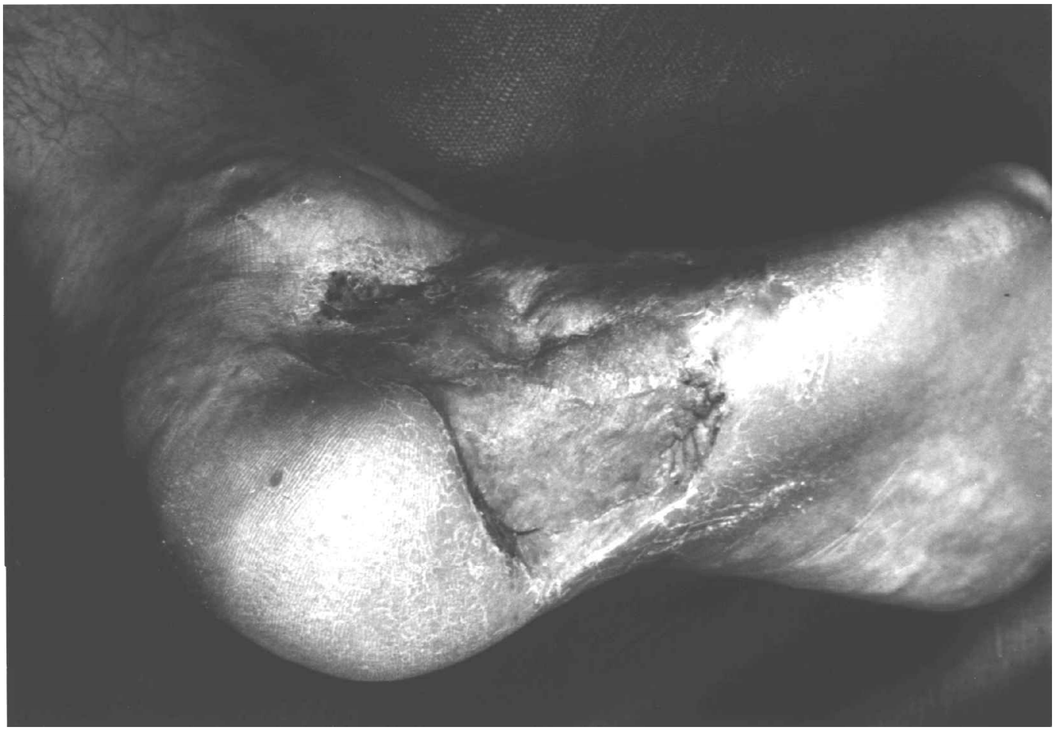

**Fig. 5.** *In order to preserve function, this young man underwent intracapsular excision of an epitheloid sarcoma from the heel. A dose of 60 Gy in 2-Gy fractions was given with two lateral fields with sparing of the sole. Local recurrence developed in the sole 3 years later, and was widely excised. A further dose of 60 Gy was delivered with a single electron beam. He remains free of disease 10 years later, is able to ski, and is pain free.*

sarcoma arising in the head and neck.[22] In our small series, positive surgical margins were not associated with significant reduction in local control following post-operative radiotherapy and a similar conclusion has been reached by others.[10]

In our series patients treated with post-operative radiotherapy following surgery for locally recurrent sarcoma had a higher rate of local failure compared to those treated at initial presentation. This suggests the need for routine post-operative radiotherapy at the time of initial conservative surgery for hand and foot sarcoma. The skin of both the palm and sole tolerates radiation poorly. However, acute skin reactions in our patient group were acceptable, with only 2 patients treated to large volumes requiring interruption of irradiation. Radiotherapy was well tolerated in the 6 patients with skin grafts who suffered no late complications. Grade 3 late skin toxicity in the form of marked atrophy and telangiectasia developed in only 5.9% of the whole series. Grade 3 late toxicity was not observed in subcutaneous tissue, bone or joints. These toxicity rates are comparable to those previously reported in the literature. Post-radiotherapy functional status was acceptable, with only 3 patients having major dysfunction which was at least partly due to surgery. No patient required amputation for

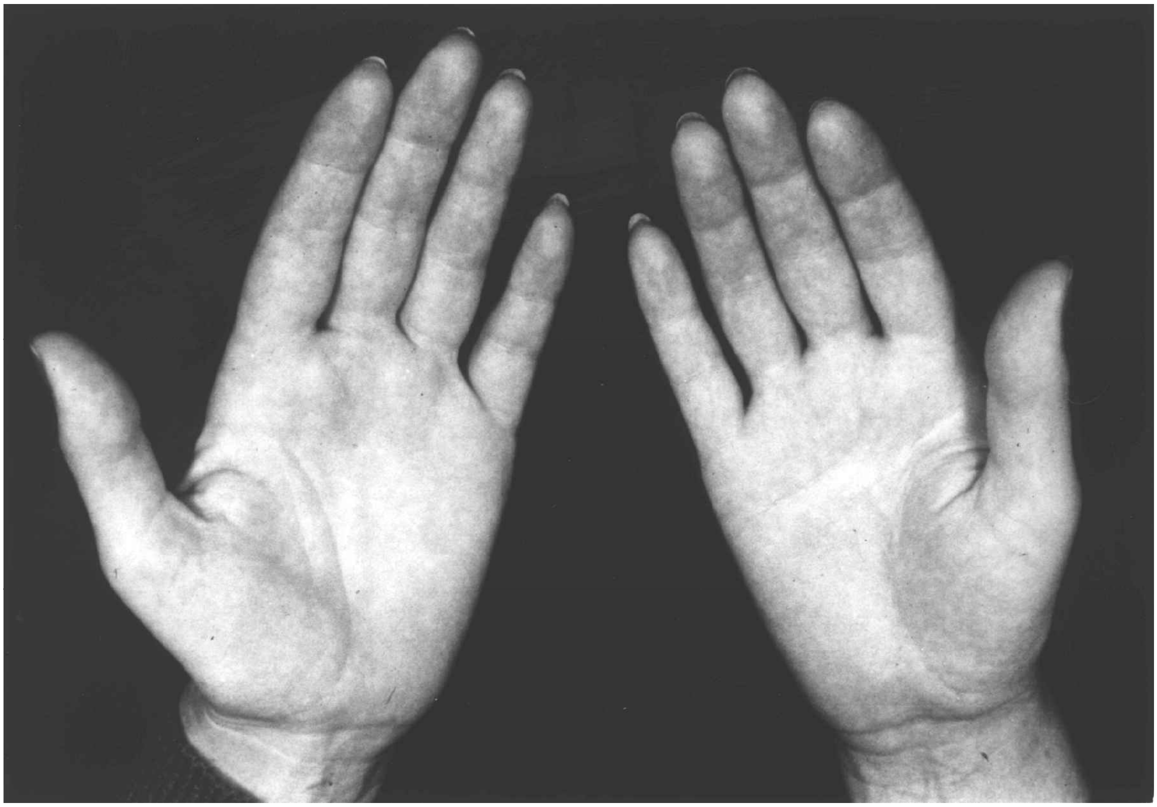

**Fig. 6**. *This young lady is a casino pit boss requiring manual dexterity and attractive looking hands in order to continue in her employment. She underwent local excision of a recurrent epitheloid sarcoma from the palm of her right hand. A dose of 60 Gy was delivered using a single direct electron field of 10 Mev energy, in 30 daily fractions, with wax build up. Moist desquamation demanded an interruption of treatment, which was completed in a total of 53 days. She remains free of disease with normal function 13 years later.*

**Table 5**. *Results of conservative surgery and post-operative radiotherapy for hand and foot sarcoma*

| Author and Year | Number of patients Hand | Foot | Local control (%) |
|---|---|---|---|
| Okunieff *et al.* (1986)[8] | 17 | 0 | 14/17 (87.0) |
| Talbert *et al.* (1990)[10] | 39 | 39 | 57/78 (74) |
| Selch *et al.* (1990)[9] | — | 20 | 17/20 (85.0) |
| Colterjohn *et al.* (1997)[12] | 0 | 25 | 24/25 (96.0) |

intractable late complication following radiotherapy. There was no difference in the functional outcome between patients with sarcoma arising in the hand compared with those arising in the foot. However, another series did report worse functional outcome in foot sarcomas which might relate to weight bearing.[10]

The local control rate in our series is low at 60%, compared to previous reports (Table 5). This may be due to the high proportion of high-grade tumours (91%) in this patient group. In addition we had a large proportion of patients treated for locally recurrent tumour (24%) compared to other series. Patients treated with radiotherapy at local recurrence have low local control rates compared to those treated after the initial surgery. This suggests the need for the routine use of post-operative radiotherapy in these tumours after initial surgery. The majority of patients in our series received a total dose of 60 Gy in 2-Gy fractions, compared to higher total doses in other series (median 65 Gy). The proportion of recurrences within the Phase II volume in patients with positive margins suggests the need for dose escalation in this patient group, possibly with conformally shaped fields. Only 11 patients in this series underwent a standard operation in the form of wide local excision prior to radiotherapy, and this may have contributed to the poor local control.

The majority of local recurrences were salvaged by further surgery. Only 6 patients required amputation for local recurrence, of whom 1 recurred on four occasions. The use of post-operative radiotherapy delayed amputation in this patient group.

Distant metastasis remains the major cause of treatment failure and death, especially in high-grade sarcoma. In our series, all patients who died of disease had evidence of uncontrollable distant metastases. The use of a conservative approach avoided mutilating surgery in these patients. The role of adjuvant chemotherapy in hand and foot sarcoma requires further evaluation but lack of effective agents is common to sarcoma at all sites.

### Conclusion

Conservative surgery followed by radiotherapy appears to be an effective treatment for soft tissue sarcoma of the hand or foot. Post-operative radiotherapy to a dose of 55–60 Gy is well tolerated, with a low incidence of moderate or severe late toxicity. The anatomical location of sarcomas in the hand or foot should not preclude the use of radiotherapy. Radiotherapy results in long-term local control in a significant proportion of patients. Amputation may be required for a small proportion of patients with isolated multiple local recurrences following radiotherapy.

### Acknowledgements

We would like to thank all the surgeons who have been kind enough to refer patients to us for radiotherapy, in particular Mr Meirion Thomas. We are most grateful to our colleagues in the departments of Physics, Radiotherapy and Radiology and also the departments of Photography at Fulham Road and Sutton.

### References

1 Stinson SF, Delaney TF, Greenberg J, *et al.* Acute and long term effects on limb function of combined modality limb sparing therapy for extremity soft tissue sarcoma. *Int J Radiat Oncol Biol Phys* 1991; 21:1493–9.

2 Brennan MF. Management of extremity soft tissue sarcoma. *Am J Surg* 1989; 158:71–8.

3 Bray PW, Bell RS, Bowen CV, *et al.* Limb salvage surgery and adjuvant radiotherapy for soft tissue sarcomas of the forearm and hand. *J Hand Surg Am* 1997; 22:495–503.

4 Owens JC, Shiu MH, Smith R, *et al.* Soft tissue sarcomas of the hand and foot. *Cancer* 1985; 55:2010–8.

5 Campanacci M, Bertoni F, Laus M. Soft tissue sarcoma of the hand. *Italian J Orthotraumatol* 1981; 7:313–27.

6 Markhede G, Angervall L, Stener B. A multivariate analysis of prognosis after surgical treatment of malignant soft tissue tumours. *Cancer* 1982; 49:1721–33.

7 Kinsella TJ, Loeffler JS, Fraass BA, *et al.* Extremity preservation by combined modality therapy in sarcomas of the hand and foot: an analysis of local control, disease free survival and functional result. *Int J Radiat Oncol Biol Phys* 1983; 9:1115–9.

8 Okunieff P, Suit HD, Proppe KH. Extremity preservation by combined modality treatment of sarcomas of the hand and wrist. *Int J Radiat Oncol Biol Phys* 1986; 12:1923–9.

9 Selch MT, Kopald KH, Ferreiro GA, *et al.* Limb salvage therapy for soft tissue sarcomas of the foot. *Int J Radiat Oncol Biol Phys* 1990; 19:41–8.

10 Talbert ML, Zagars GK, Sherman NE, *et al.* Conservative surgery and radiation therapy for soft tissue sarcomas wrist, hand, ankle and foot. *Cancer* 1990; 66:2482–91.

11 Johnstone PA, Wexler LH, Venzon DJ, *et al.* Sarcomas of the hand and foot: Analysis of local control and functional result with combined modality therapy in extremity preservation. *Int J Radiat Oncol Biol Phys* 1994; 29:735–45.

12 Colterjohn NR, Davis AM, O'Sullivan B, *et al.* Functional outcome in limb salvage surgery for soft tissue tumours of the foot and ankle. *Sarcoma* 1997; 1:67–74.

13 Le Vay J, O'Sullivan B, Catton C, *et al.* Outcome and prognostic factors in soft tissue sarcoma in adult. *Int J Radiat Oncol Biol Phys* 1993; 27:1091–9.

14 Lindberg RD, Martin RG, Romsdahl MM, Barkley HT. Conservative surgery and post operative radiotherapy in 300 adults with soft tissue sarcomas. *Cancer* 1981; 47:2391–7.

15 Lawrence W, Hays DM. Surgical lessons from the Intergroup Rhabdomyosarcoma Study. *NCI Monogr* 1981; 56:159–63.

16 Marcove RC, Rosen G. Radical en block excision of Ewing's sarcoma. *Clin Orthoped Related Res* 1980; 153:86–91.

17 Simon MA, EnneKing WF. The management of soft tissue sarcomas of the extremities. *J Bone Joint Surg* 1976; 58A:317–27.

18 Harmer C, Bidmead M. Three dimensional planning and conformal radiotherapy In: Verweij J, Pinedo HM, Suit HD, eds. *Soft tissue sarcomas: present achievements and future prospects* (Kluwer, 1997; 129–41).

19 Robinson MH, Cassoni A, Harmer CL, Thomas JM, Westbury G. High dose hyperfractionated radiotherapy in the treatment of extremity soft tissue sarcomas. *Radiother Oncol* 1991; 22:118–26.

20 Collin CF, Friedrich C, Godbolld J, Hadju S, Brennan MF. Prognostic factors for local recurrence and survival in patients with localized extremity soft tissue sarcoma. *Semin Surg Oncol* 1988; 4:30–7.

21 Bell RS, O'Sullivan B, Lin FF, *et al.* The surgical margin in soft tissue sarcoma. *J Bone Joint Surg* 1989; 71:370–5.

22 Eeles RA, Fisher C, A'Hern RP, *et al.* Head and neck sarcomas: prognostic factors and implications for treatment. *Br J Cancer* 1993; 68:201–7.
